# The annexin A1/FPR2 signaling axis expands alveolar macrophages, limits viral replication, and attenuates pathogenesis in the murine influenza A virus infection model

**DOI:** 10.1096/fj.201901265R

**Published:** 2019-10-02

**Authors:** Sebastian Schloer, Nicole Hübel, Dörthe Masemann, Denise Pajonczyk, Linda Brunotte, Christina Ehrhardt, Lars-Ove Brandenburg, Stephan Ludwig, Volker Gerke, Ursula Rescher

**Affiliations:** * Center for Molecular Biology of Inflammation, Institute of Medical Biochemistry, University of Muenster, Muenster, Germany;; † Cells-in-Motion Cluster of Excellence, University of Muenster, Muenster, Germany;; ‡ Center for Molecular Biology of Inflammation, Institute of Virology, University of Muenster, Muenster, Germany;; § Section for Experimental Virology, Institute of Medical Microbiology, Jena University Hospital, Jena, Germany;; ¶ Department of Anatomy and Cell Biology, RWTH Aachen University, Aachen, Germany;; ‖ Institute of Anatomy, Rostock University Medical Center, Rostock, Germany

**Keywords:** pattern recognition receptors, innate immune system, mucosal immunity

## Abstract

Pattern recognition receptors (PRRs) are key elements in the innate immune response. Formyl peptide receptor (FPR) 2 is a PRR that, in addition to proinflammatory, pathogen-derived compounds, also recognizes the anti-inflammatory endogenous ligand annexin A1 (AnxA1). Because the contribution of this signaling axis in viral infections is undefined, we investigated AnxA1-mediated FPR2 activation on influenza A virus (IAV) infection in the murine model. AnxA1-treated mice displayed significantly attenuated pathology upon a subsequent IAV infection with significantly improved survival, impaired viral replication in the respiratory tract, and less severe lung damage. The AnxA1-mediated protection against IAV infection was not caused by priming of the type I IFN response but was associated with an increase in the number of alveolar macrophages (AMs) and enhanced pulmonary expression of the AM-regulating cytokine granulocyte-M-CSF (GM-CSF). Both AnxA1-mediated increase in AM levels and GM-CSF production were abrogated when mouse (m)FPR2 signaling was antagonized but remained up-regulated in mice genetically deleted for mFPR1, an mFPR2 isoform also serving as AnxA1 receptor. Our results indicate a novel protective function of the AnxA1-FPR2 signaling axis in IAV pathology *via* GM-CSF–associated maintenance of AMs, expanding knowledge on the potential use of proresolving mediators in host defense against pathogens.—Schloer, S., Hübel, N., Masemann, D., Pajonczyk, D., Brunotte, L., Ehrhardt, C., Brandenburg, L.-O., Ludwig, S., Gerke, V., Rescher, U. The annexin A1/FPR2 signaling axis expands alveolar macrophages, limits viral replication, and attenuates pathogenesis in the murine influenza A virus infection model.

The respiratory tract is constantly exposed to foreign particles and is, therefore, protected against inappropriate inflammatory activation and associated tissue damage. Through their high phagocytic activity and a low response to inflammatory stimuli, alveolar macrophages (AMs) that reside in the bronchoalveolar lumen are key players in maintaining a tolerogenic environment (1). AMs are the first cells that encounter pathogens, and the disappearance of this cell population is usually observed during infection and inflammation, including influenza A virus (IAV) infection.

Regularly, IAV infection manifests in mammals only in the respiratory tract in humans and animals (2, 3), with the majority of infected people fully recovering within a few weeks, even without medical treatment. However, in risk groups, including young, elderly, and immune-suppressed patients, IAV infection can lead to severe complications (*e.g.*, pneumonia and death) (4). Thus, IAV infections are among the most dangerous global virus-related diseases and are a major public health concern. The critical role of AMs in the host defense against IAV infection has been demonstrated in a range of *in vivo* infection models (5). In IAV-infected mice, depletion of AMs and an accompanying deficiency of granulocyte-M-CSF (GM-CSF), the cytokine that supports AM homeostasis, is observed (6, 7). AMs play a decisive role in the functional maintenance of the IAV-infected lung (8). AM depletion prior to IAV infection increases the severity of IAV disease, whereas GM-CSF administration or adoptive transfer of AMs is beneficial (7, 9). Thus, the identification of immune mediators and pathways that control the lung inflammatory immune response *via* AM activation might unravel novel drug targets for the prophylaxis and the prevention of lung pathology.

Key elements in the innate immune response are pattern recognition receptors (PRRs) that detect and respond to conserved molecular structures found across a broad range of entire classes of related pathogens (10). Under pathophysiological conditions, however, host cells release components that are also recognized by PRRs and consequently trigger innate immune responses. In line with this notion, ligands of the formyl peptide receptor (FPR) subfamily of PRRs comprise structurally very diverse classes of compounds, ranging from small, formylated bacterial peptides to various endogenous ligands such as annexin A1 (AnxA1), a 37 kDa protein of the annexin protein family and probably one of the best known endogenous agonists for FPRs (11, 12). The physiologic relevance of the AnxA1-mediated immune regulation *via* activation of the FPR signaling axis has been confirmed in a vast body of *in vivo* studies utilizing FPR knockout (KO) mice as well as FPR antagonists (13–15). Because increased levels of AnxA1 (12) are found in bronchoalveolar lavage (BAL) fluids of a range of pathologic conditions, such as lung cancer, chronic obstructive pulmonary disease, and bronchial asthma (12, 16–18), we addressed whether FPR activation *via* AnxA1 impacts the generation or activation of AMs. Here, we report a novel function of the AnxA1-FPR axis. Mice treated with AnxA1 displayed enhanced expression of GM-CSF and increased numbers of AMs in the lung. Furthermore, AnxA1 treatment limited subsequent IAV replication and protected against IAV-associated pathology and morbidity. Mechanistically, the protective function was coupled specifically to FPR2 activation. Our study provides evidence for a pivotal impact of the AnxA1-FPR signaling axis on AM-mediated protection against IAV.

## MATERIALS AND METHODS

### Drug treatment and *in vivo* IAV infection of mice

Human recombinant AnxA1 was expressed as previously reported (19). The absence of endotoxin was evaluated with the LAL Chromogenic Endotoxin Quantitation Kit (Thermo Fisher Scientific, Waltham, MA, USA; Pierce, Rockford, IL, USA) and was routinely below 0.1 EU/ml. The FPR2 antagonist WRW4 was obtained from Tocris (Tocris Bioscience, Bristol, United Kingdom).

WT C57BL/6 mice were purchased from Envigo (Huntingdon, United Kingdom). The constitutive FPR1 KO mice were kindly provided by Lars-Ove Brandenburg, (RWTH Aachen University and Rostock University Medical Center, Rostock, Germany). Animals were kept under specific pathogen-free conditions. All experiments were approved by the State Agency for Nature, Environment and Consumer Protection North Rhine-Westphalia (LANUV; 84-02.04.2015.A049) and were performed in compliance with the guidelines for the welfare of experimental animals issued by the Federal Government of Germany and the state of North Rhine-Westphalia.

Eight- to ten-week-old male animals were intraperitoneally injected with 20 µg/kg body weight of recombinant human AnxA1 diluted in PBS or 7.5 mg/kg body weight WRW4. Dosages were selected based on previous studies (20–22). We further analyzed the biologic activity of AnxA1 by measuring the Ca^2+^ release capacity in HeLa-FPR2 cells prior to the application to animals (control: inhibition of Ca^2+^ release by using the FPR2 antagonist WRW4) (Supplemental Fig. S1*A*). Control animals received the equivalent volume of sterile saline 4 d prior to infection with IAV. Mice were anesthetized with ketamine-xylazine (0.5% K, 0.1% X, 10 ml/kg body weight) prior to intranasal infection with IAV strain PR8 [A/Puerto Rico/8/1934(H1N1)]. To assess pain and distress during the course of infection, animals were assessed based on a scoring system with sufficiently frequent observation times that assigns numerical values to several criteria of animal conditions that were considered signs of morbidity or moribundity, including changes in body temperature, physical appearance, behavior, and weight loss. Animals that reached the cumulative threshold score were euthanized. A body weight loss of >20% compared with start of the treatment was the cutoff parameter for euthanasia, regardless of the total score. The date of death for euthanized mice was marked as the date of euthanasia. None of the animals died during the course of the experiment.

### Validation of AnxA1 and WRW4 activity

AnxA1 and WRW4 biologic activities were validated using HeLa cells stably expressing FPR2 (23) *via* ratiometric fluorescence microscopy imaging of Ca^2+^ signals. Cells were pulsed for 30 min with the Ca^2+^-sensitive dyes Fluo-4-AM (Fluo4, ABD-20551; Biomol/ATT Bioquest, Hamburg, Germany) and Fura Red-AM (Biomol/ATT Bioquest). Cells were washed and kept in 4-(2-hydroxyethyl)-1-piperazineethanesulfonic acid–buffered Hank's balanced salt solution at 37°C during image acquisition on a Zeiss LSM 780 microscope (Oberkochen, Germany). Epifluorescence signal was acquired for each of the dyes individually and at intervals of 1 s, and ratio values of Fluo4/FuraRed fluorescence were calculated. Treatment-induced increases in cytosolic Ca^2+^ were calculated as percentage of maximum increase induced with 500 nM ionomycin.

### *In vitro* infection assay of primary mouse lung cells

Lungs of AnxA1- and control-treated mice were extracted and digested as previously described in Heitzig *et al*. (24). Primary mouse lung cells were then infected with PR8 (MOI 0.01), and the supernatants were harvested at indicated time points.

### Plaque titration

Tissue from infected mice was harvest at various time points postinfection (p.i) (0, 1, 3, 5, or 7 d p.i.) and homogenized utilizing Lysing Matrix (MD Biomedicals, Santa Ana, CA, USA) according to the manufacturer’s protocol. Amounts of infectious particles in the supernatants of tissue homogenates or infected cells were determined by a standard plaque assay technique using confluent MDCK cells as published in Musiol *et al*. (25).

### Lung histology

Isolated lungs tissues were fixed for 4 h in 4% buffered formaldehyde solution (pH 7.4), dehydrated in a series of graded alcohols, and embedded in paraffin. To assess the degree of tissue inflammation, hematoxylin and eosin–stained 4-μm sections were examined using a Keyence BZ-9000 microscope (Osaka, Japan). Tissue densification as an indicator of immune cell invasion and fibrosis was quantified using BZ-II Analyzer software (Keyence).

### Real-time quantitative PCR

Murine lungs were homogenized in RLT buffer (Qiagen, Germantown, MD, USA) supplemented with 1% 2-ME using lysing matrix tubes (MD Biomedicals). Total RNA was isolated with the RNeasy Mini Kit (Qiagen) according to the manufacturer’s instructions, and 1 µg was converted into cDNA using the High-Capacity cDNA Reverse Transcription Kit and random primers (Thermo Fisher Scientific). Antiviral gene expression in the lung was analyzed by SYBR green quantitative PCR (qPCR) (Platinum SYBR Green qPCR SuperMix-UDG w/Rox; Thermo Fisher Scientific) using QuantiTect Primer Assays (Qiagen Mm_Mx1_3_SG, QT02329236; Mm_Mx2_1_SG, QT00106743; Mm_Oasl1_1_SG, QT00128891; Mm_Irf7_1_SG, QT00245266; Mm_Anxa1_1_SG, QT00145915) on a CFX 384 Real-Time PCR Cycler with the CFX Manager Software v.2.1 (Bio-Rad, Hercules, CA, USA), with the expression values normalized to house-keeping genes glyceraldehyde-3-phosphate dehydrogenase (GAPDH; Mm_Gapdh_3_SG, QT01658692), β-actin (Mm_Actb_1_SG, QT00095242), and b-2 microglobulin (Mm_B2m_2_SG, QT01149547). CSF expression was evaluated using TaqMan primer/probe sets for murine GM-CSF, G-CSF, and M-CSF from the Universal ProbeLibrary (Roche, Basel, Switzerland) on a LightCycler 480 Instrument II (Roche), with the expression values normalized to GAPDH and cytochrome c. Samples from independent experiments were run in triplicates. For analysis of changes in gene expression, the ΔΔ*C_t_* method was used. In brief, *C_t_* values for genes of interest in the individual samples were normalized to the housekeeping genes, resulting in Δ*C_t_* values. Mean Δ*C_t_* values were calculated, and ΔΔ*C_t_* values were obtained by subtraction. 2^−ΔΔ^*^Ct^* was used to calculate the relative fold gene expression levels. One-way ANOVA on ΔΔ*C_t_* values was used to analyze the statistical significance of the differences.

### Analysis of BAL fluids

BAL was performed by flushing the lungs of euthanized mice 3 times with 700 µl PBS/2 mM EDTA at 4°C. Total protein concentrations in the BAL fluids were quantified using the Coomassie Protein Assay Kit (Pierce). Lactate dehydrogenase (LDH) levels were determined using the *in vitro* Toxicology Assay Kit (TOX7; MilliporeSigma, Burlington, MA, USA) according to the manufacturer’s protocol.

### Cytokine measurements

Analysis of IFN-γ, IL-1β, IL-4, IL-6, monocyte chemoattractant protein (MCP)-1, and macrophage inflammatory protein (MIP)-1β levels in the BAL fluids was carried out using the Cytometric Bead Array Flex Sets (BD Biosciences,San Jose, CA, USA) according to the manufacturer’s instructions, with a FACS Calibur cytometer and the CellQuestPro and FCAP Array v.3.0 Software (BD Biosciences). Murine IFN-λ was measured using the ELISA Kit from PBL Assay Science (Piscataway, NJ, USA) according to the manufacturer’s instructions.

### Flow cytometry and determination of infiltrating leukocytes

Cells in the BAL fluids were fixed *via* FACS Cell Fixation Buffer (eBioscience, San Diego, CA, USA), blocked with mouse Seroblock FcR (1 µg for 10^6^ cells/100 µl; Bio-Rad) for 10 min at 4°C, and stained with the appropriate antibodies for 30 min at 4°C. Flow cytometry and data analysis were performed using a Guava easyCyte12 Cytometer and the InCyte Software (MilliporeSigma). AMs, blood-derived monocytes/macrophages, and neutrophils were gated as indicated using antibodies against murine CD11c (clone N418), CD45 (cone I3/2.3), and I-A/I-E (clone M5/114.15.2) from BioLegend (San Diego, CA, USA), antibody against murine CD11b (clone M1/70) and Ly-6G (clone RB6-8C5) from eBioscience, and antibodies against murine CD24 (clone M1/69) and Siglec-F (clone E50-2440) from BD Biosciences.

### Rhodamine B-dextran uptake

Quantitation of AM endocytosis was performed essentially as described in Sallusto *et al*. (26). In brief, 2 × 10^6^ cells/ml were incubated in VLE-RPMI medium (Biochrom, Cambridge, United Kingdom) supplemented with 10% fetal calf serum (Biochrom), 1% l-glutamine (Lonza, Basel, Switerland), 100 U/ml penicillin [phenylacetic acid (PAA)], 100 µg/ml streptomycin (PAA), 10 mM 4-(2-hydroxyethyl)-1-piperazineethanesulfonic acid (AppliChem, Darmstadt, Germany), and 1 mg/ml rhodamine B-dextran (MW 70000; Thermo Fisher Scientific) at 37°C for 120 min. Unspecific background signals were determined from cells incubated for the same period of time at 4°C and subsequently subtracted. Cells were washed with ice-cold PBS and 5% bovine serum albumin/CellWash (BD Biosciences), blocked for 10 min at 4°C with mouse Seroblock FcR (AbDSerotec) with anti-mouse antibodies used to identify AMs (CD45^+^CD11b^+^Siglec-F^+^) for 30 min at 4°C, washed 2 times with 5% bovine serum albumin/CellWash, and analyzed using the Guava easyCyte12 Cytometer and the InCyte Software (MilliporeSigma).

### Statistical analysis

*A priori* power analysis (G*Power 3.1; Universität Düsseldorf, Düsseldorf, Germany) (27) was used to estimate required sample sizes. Data were analyzed with Prism 6.00 (GraphPad Software, La Jolla, CA, USA). Statistically significant differences were evaluated using the Mantel-Cox log rank test and Mann-Whitney *U* test.

## RESULTS

### AnxA1 treatment attenuates IAV-induced pathology *in vivo*

**Figure 1:**
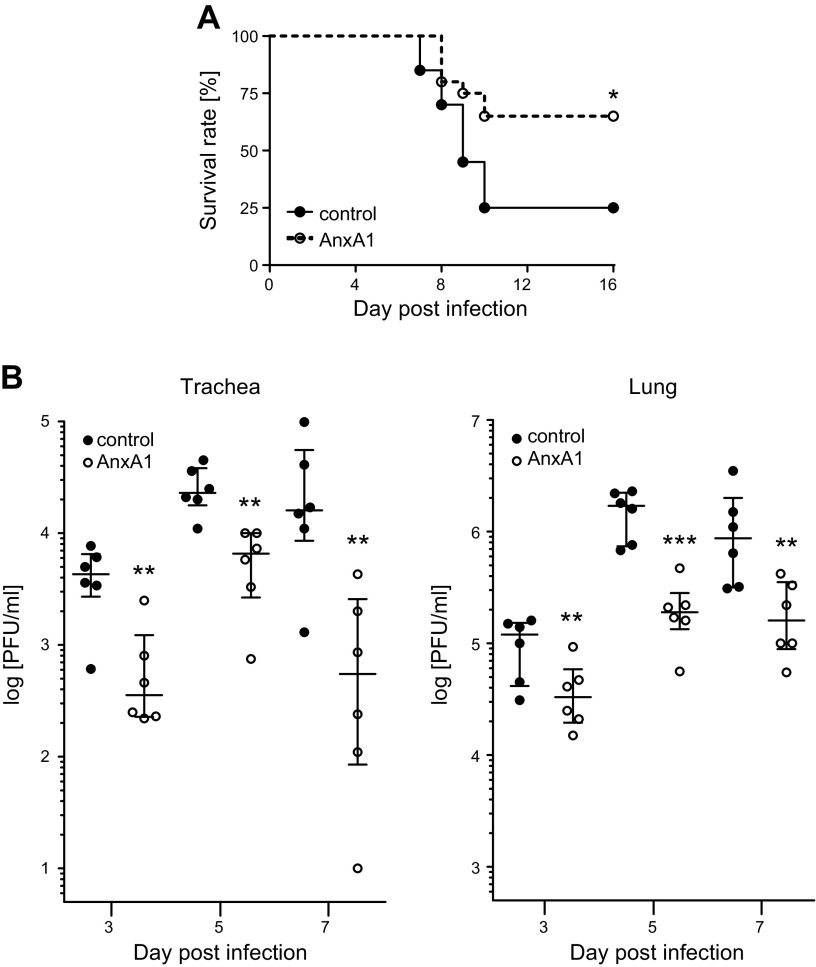
AnxA1 treatment protects against subsequent IAV infection. Control or AnxA1-treated mice were infected intranasally with 500 pfu IAV H1N1 strain PR8 4 d post AnxA1 treatment (d 0). *A*) Cumulative survival rate of AnxA1- or control-treated mice at the indicated days p.i. Mortality also includes mice that were euthanized because of a body weight loss of ≥20%; *n* = 20 mice/group, Mantel-Cox log rank test, *P* = 0.0136. *B*) Viral loads in trachea and lungs of the individual mice at indicated times p.i. Data are expressed as scatter plots of individual mice data with the medians and the interquartile ranges superimposed; *n* = 6 mice/group. **P < 0.01, ***P < 0.001 (Mann-Whitney *U* test).

To assess the impact of AnxA1 treatment on viral infections, mice were administered a single dose of AnxA1. Four days later, the mice were infected intranasally with IAV strain PR8 [A/Puerto Rico/8/34(H1N1)] and subsequently monitored until d 16 p.i. Strikingly, a markedly reduced mortality was observed in the AnxA1-treated group (65% survival rate) compared with the control group (25% survival rate) during the course of IAV infection (**Fig. 1*A***).

To further investigate the protective function of AnxA1 treatment on IAV infection outcome, we next determined the viral burden in trachea and lung homogenates. Plaque assays revealed significantly lower virus levels in AnxA1-treated mice (Fig. 1*B*), suggesting that AnxA1 treatment prior to infection counteracted viral propagation.

**Figure 2:**
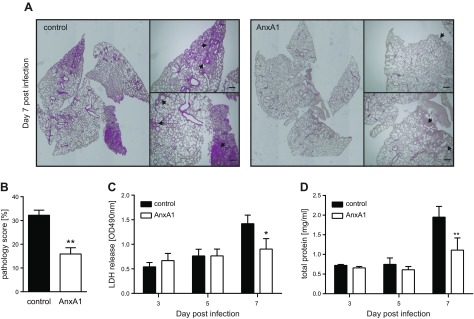
AnxA1 treatment attenuates IAV-induced lung damage. *A*) Histopathological analysis of lungs of AnxA1-treated or control animals on d 7 p.i. Hematoxylin and eosin lung sections are shown. Higher magnifications of boxed regions are shown for comparison of inflammatory signs. Scale bars, 200 µm. *B*) To score lung inflammation, separate sections of each lung were evaluated for tissue density, and the mean density/lung was calculated and compared by Mann-Whitney *U* test (*n* = 3). *C*, *D*) LDH activity in the BAL (*C*) and concentration of total protein in the BAL (*D*) are depicted. Each bar represents the mean ± sem of 8 mice at the indicated day p.i. *P < 0.1, **P < 0.001 (Mann-Whitney *U* test).

Because virus-induced lung injury is a major factor in IAV-related morbidity and mortality (28, 29), we next analyzed whether the restricted viral replication observed in AnxA1-treated animals correlated with lower levels of lung damage. Histopathological analysis of the respective lung sections revealed that upon infection, both groups displayed inflamed regions typically observed during IAV infection. However, severe inflammatory cell infiltrates and inflammatory foci were detected in the alveolar parenchyma of control mice at d 7 p.i, whereas inflammation was moderate and did not encroach on the parenchyma in lungs obtained from AnxA1-treated mice (**Fig. 2*A***). Quantitative scoring of lung injury confirmed that the lungs of AnxA1-treated mice were indeed significantly less affected compared with the control group at d 7 p.i. (Fig. 2*B*). Consistently, AnxA1-treated mice presented lower LDH and total protein levels in the cell-free BAL fluids (Fig. 2*C, D*), indicating less disturbed cellular integrity and alveolar leakage. Collectively, these data indicate that the reduced lung damage caused by lower virus titers accounts for the beneficial effect of AnxA1 treatment. Interestingly, we observed a tendency toward up-regulation of AnxA1 mRNA in AnxA1-pretreated and IAV-infected mice on d 7, indicative of an enhancement of the protective effect *via* an autocrine mechanism, as previously observed in Rescher *et al*. (30). (Supplemental Fig. S1*C*).

**Figure 3:**
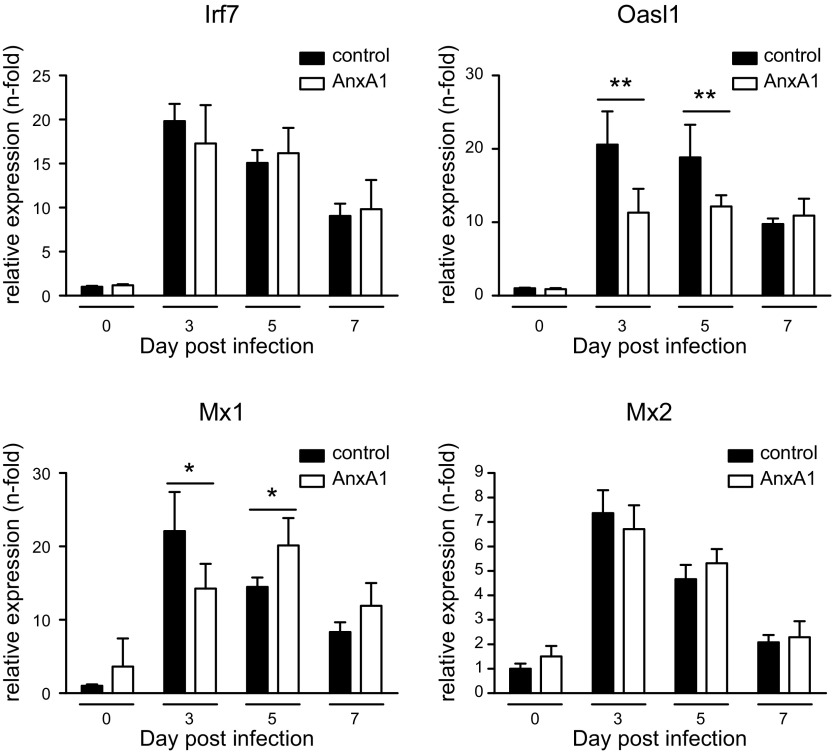
Expression of IFN-induced antiviral genes in AnxA1-treated mice. Lungs of AnxA1 or nontreated animals were analyzed during the course of IAV infection for the expression levels of the indicated IFN-stimulated genes by qPCR. Bar graphs represent relative expression levels ± sem relative to the 3 reference genes GAPDH, actin β (ACTB), and β₂ macroglobulin (B2M) at the indicated day p.i; *n* = 5 mice/group. *P < 0.1, **P < 0.001 (Mann-Whitney *U* test).

**Figure 4:**
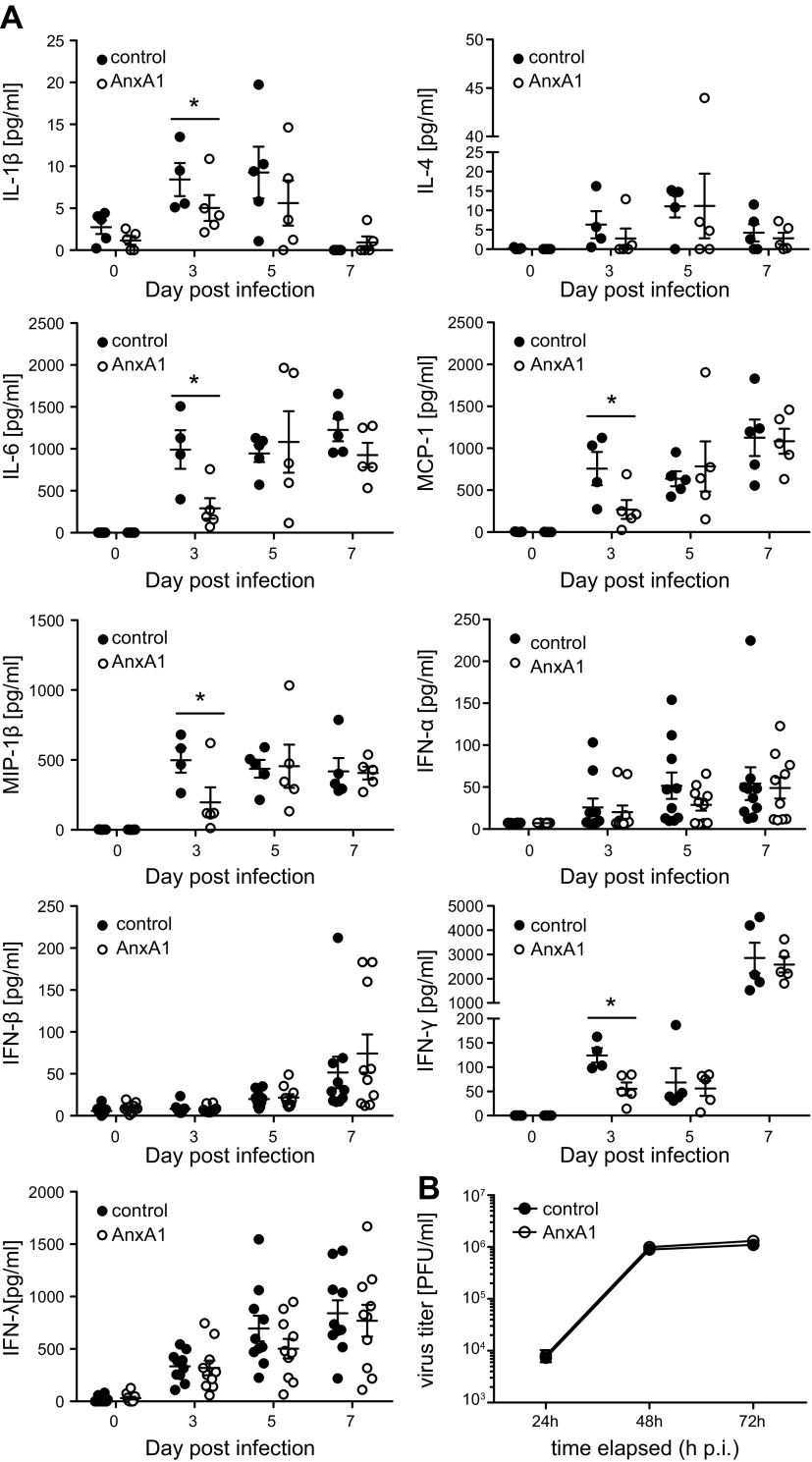
Cytokine release in virus-infected mouse lungs is not significantly altered during the course of infection. *A*) BAL fluids were obtained at indicated days p.i. Levels of the indicated cytokines were determined by flow cytometry–based multiplex bead assay or ELISA. Bars represent mean values ± sem; *n* = 5–8 mice/group. *B*) Murine primary lung cells were treated with AnxA1 or with the vehicle and subsequently infected with PR8 (multiplicity of infection, 0.01) for the indicated period of time. Viral load was determined by plaque assay. *P < 0.05 (Mann-Whitney *U* test).

To further elucidate the protective effect of AnxA1, we next examined the type I IFN response at the mRNA and protein level. As revealed by qPCR, expression of representative type I IFN-induced genes was similar in the lung homogenates of AnxA1-treated and control animals, indicating that AnxA1 did not evoke an IFN-mediated antiviral state. Upon IAV infection, AnxA1-treated animals trended toward a reduced induction of antiviral genes at d 3 p.i. (**Fig. 3**), which most likely reflected the reduced viral burden observed in these animals. Similarly, BAL fluids revealed a trend toward lower cytokine levels in AnxA1-treated mice infection. This was most evident for the ILs IL-1β and IL-6 as well as MCP-1 and MIP-1, which was significantly lower in BAL fluids obtained from AnxA1-treated and subsequently IAV-infected animals (**Fig. 4*A***). To further elucidate the mechanism underlying reduced viral replication upon AnxA1 treatment, we checked viral infection efficacy and replication properties in murine primary epithelial lung cells. Notably, primary cells derived from lungs of control and AnxA1-treated mice were equally susceptible to infection by IAV (Fig. 4*B*), indicating that the treatment did not induce an IFN-induced antiviral state.

### AnxA1-mediated antiviral protection is associated with increased numbers and activation state of AMs

**Figure 5:**
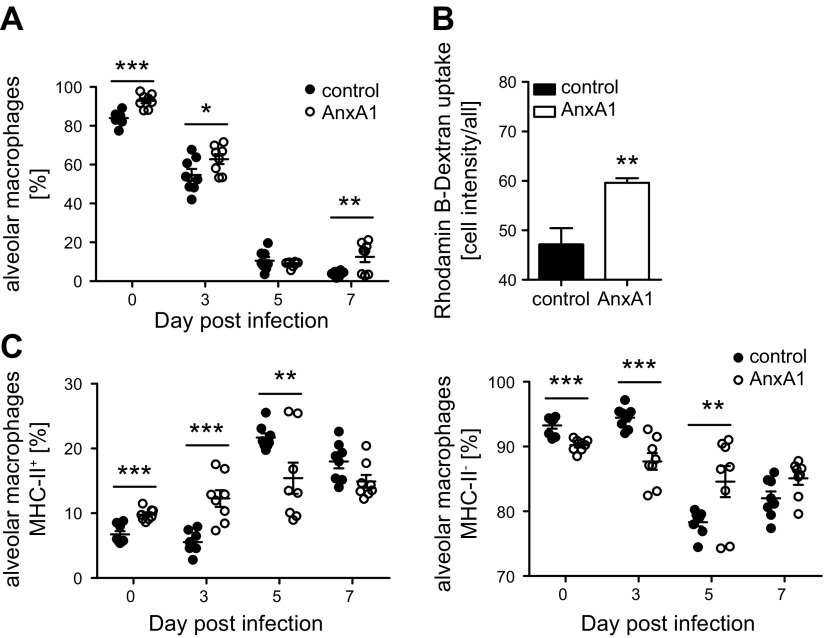
AnxA1 treatment expands and activates AMs. Cells in BAL fluids of IAV-infected mice obtained at the indicated days of treatment were pregated on CD45^+^ events and were analyzed for the presence of subsets. *A*) AMs (CD11c^+^Siglec-F^+^) were expressed as percentages among all CD45^+^ cells. *B*) Rhodamine B–labeled dextran was added to BAL for 120 min. AMs were identified and analyzed for dextran uptake by flow cytometry. Bar graphs show median fluorescence intensities ± sem of 5000 cells/BAL; *n* = 5 mice/group. *C*) AMs were further analyzed for the frequencies of MHCII^+^ or MHCII^−^ cells; *n* = 8 mice/group. Symbols represent values of individual mice, with the means ± sem superimposed. **P < 0.01. ***P < 0.001 (Mann-Whitney *U* test).

Because our observations did not indicate AnxA1-induced priming of the type I IFN response in uninfected animals, we hypothesized that the protective function on IAV mortality might be evoked through the generation or activation of specific immune cells that function in the first line of antiviral defense. Key elements of the lung immune system include AMs that develop from lung-resident progenitor cells independently of bone marrow–derived monocytes (31). *In situ*, AMs proliferate at a slow rate (32) and exert a critical protective effect against IAV (33, 34). Thus, mice were treated with AnxA1, and the BAL fluids were analyzed for the presence of AMs 4 d later by multicolor flow cytometry utilizing CD45 as a pan-leukocyte marker (35) and a gating strategy as shown in Supplemental Fig. S1*B*. Importantly, we found that the percentage of AMs was significantly elevated in the BAL fluid of AnxA1-treated mice compared with control mice (**Fig. 5*A***). Further characterization revealed that in AnxA1-treated animals, AMs presented both significantly up-regulated major histocompatibility complex class II (MHCII) cell surface expression and enhanced internalization of high-molecular dextran (Fig. 5*B, C*), suggesting an elevated activation state. At early stages of infection, the percentage of AMs in the BAL fluid of AnxA1-treated mice compared with control mice was still significantly elevated (Fig. 5). In both groups, the AM pool was subsequently depleted at d 5 p.i.; however, we observed an earlier recovery at 7 d p.i. in AnxA1-treated mice. Thus, our results argue for a protective function toward IAV-associated mortality evoked through AnxA1-FPR2–mediated AM expansion.

### GM-CSF expression is mediated *via* the AnxA1-FPR2 signaling axis

**Figure 6:**
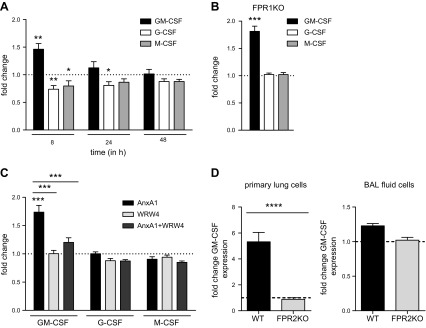
AnxA1 induces differential pulmonary expression of the colony-stimulating factors *via* FPR2. *A*) qPCR analysis of expression levels of GM-CSF, G-CSF, and M-CSF in lungs of AnxA1-treated mice compared with control animals at the indicated time points postinjection; 8 h, *n* = 8; 24 h, 48 h, *n* = 5 mice/group (Mann-Whitney *U* test). *B*). AnxA1-induced changes in expression levels detected at 8 h postinjection in lungs of FPR1 KO mice. *C*) CSF expression in mice treated either with a single dose or a combination of AnxA1 and the FPR2 antagonist WRW4. Note that WRW4 abrogates the AnxA1-mediated effect on GM-CSF up-regulation. *D*) qPCR analysis of GM-CSF expression levels in primary lung cells and BAL fluid cells treated or not for 8 h with 400 nM AnxA1. For all graphs, bars represent fold change of relative expression levels ± sem, and dashed (dotted) lines indicate no change (expression ratio = 1).

AM proliferation and differentiation are critically influenced through the CSF cytokine family, namely the balance between M-CSF and GM-CSF (31, 36). In line with an elevated AM pool, qPCR analysis revealed significantly elevated GM-CSF expression levels in lung homogenates of AnxA1-treated animals compared with control mice, whereas levels of G-CSF and M-CSF were slightly lowered. The up-regulation of GM-CSF was transient, and the levels equalized to those observed in control mice 24 h after AnxA1 treatment (**Fig. 6*A***). Because AnxA1 is a specific endogenous ligand for the chemotactic FPR subfamily of the GPCR (37, 38), we reasoned that AnxA1-mediated activation of FPRs might be responsible for the up-regulation of GM-CSF expression in lungs of AnxA1-treated mice. In the murine system, 2 main isoforms of this receptor family, mouse (m)FPR1 and mFPR2, have been shown to function similarly to their human homologs (38). To explore the involvement of the AnxA1-FPR axis, we first investigated the AnxA1-mediated CSF production in lungs from mice genetically deficient in the expression of mFPR1. As shown in Fig. 6*B*, GM-CSF expression was clearly up-regulated in mFPR1-KO mice, suggesting that the up-regulation of lung GM-CSF is independent of mFPR1 activation.

To examine whether AnxA1 triggers GM-CSF induction *via* activation of the homologous mFPR2, we next investigated the impact of mFPR2 signaling on AnxA1-mediated CSF induction. Because the constitutive lack of the FPR2 gene has recently been reported to correlate with dysfunctional production of myeloid progenitor cells in nonchallenged animals (39), we decided to functionally interfere with AnxA1-mediated mFPR2 activation by acute pharmacological inhibition *via* the widely used FPR2 antagonist WRW4 (40–42). Treatment with WRW4 only had no effect on CSF expression levels in the lungs (Fig. 6*C*). However, a combined treatment of AnxA1 and its antagonist WRW4 abrogated the effect of AnxA1 on GM-CSF up-regulation (Fig. 6*C*), indicating that GM-CSF expression was mediated *via* AnxA1-induced activation of mFPR2. GM-CSF mRNA levels were significantly increased in primary lung cells but not in BAL cells, which are predominantly AMs, upon stimulation with AnxA1 for 8 h (Fig. 6*D*).

**Figure 7:**
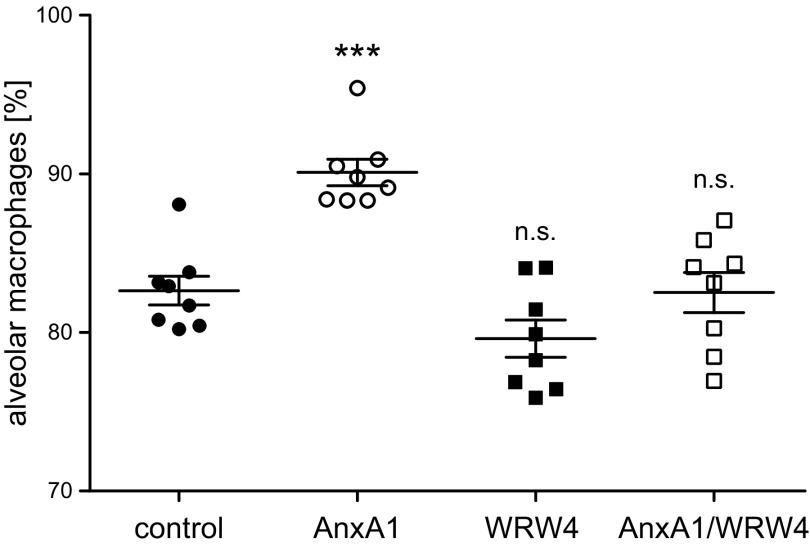
FPR2 antagonist WRW4 abrogates the AnxA1-mediated AM expansion. Cells in BAL fluids of IAV-infected mice obtained 4 d posttreatment were pregated on CD45^+^ events and analyzed for the presence of AMs (CD11c^+^Siglec-F^+^). Data show the percentage of AMs among CD45^+^ cells detected in the individual BALs. *n* = 8 mice/group, 1-way ANOVA with Sidak posttest. Differences between the control group and experimental groups are indicated; ns, nonsignificant. ***P < 0.001.

To further prove the link between the observed GM-CSF up-regulation *via* AnxA1-mediated FPR2 activation and the accompanying AM expansion, we treated mice with either vehicle, AnxA1, WRW4, or AnxA1/WRW4 and analyzed the amount of AMs found in the respective BALs. Indeed, combined treatment with AnxA1 and the FPR2 antagonist WRW4 prevented the increase in the AM pool observed upon AnxA1 treatment. Treatment with WRW4 alone does not affect the AM population (**Fig. 7**). These results strongly support the notion that AnxA1-triggered FPR2 activation and the resulting up-regulation of GM-CSF are directly associated with the increase in AMs. Thus, our results argue for a protective function toward IAV-associated mortality evoked through AnxA1-FPR2–mediated AM expansion.

## DISCUSSION

The primary targets for productive IAV replication are alveolar epithelial cells, and IAV infection is associated with extensive induction of proinflammatory mechanisms (43). We found that in AnxA1-treated animals, the increased host survival was associated with significantly decreased IAV-associated lung injury as well as strongly reduced viral titers observed as early as d 3. Consistent with this observation, the respective cytokine levels at that time point also suggest a weaker antiviral response because of reduced viral burden in these animals. During the course of infection, loss of alveolar barrier function, edema formation, and endothelial cell death by apoptosis and virus-caused cytopathogenic effects contribute to severe lung pathology, which may progress to fatal lung failure (44, 45). Indeed, the extent of IAV-induced tissue damage and the epithelial repair, leading to tissue regeneration and restoration of the barrier function, are critical factors in IAV pathogenicity (33). Untreated control animals died as of d 7, commonly seen in mice infected with PR8 at a LD_50_, and strong inflammatory signs were observed in all mice that were alive at this point in time. At earlier time points during infection, the extent of lung damage of infected control animals varied. Assuming that the reduced virus titers in AnxA1-treated mice correlate with less subsequent lung damage, we expected a difference in the extent of lung pathology to be clearly recognizable at d 7, which was indeed the case.

As IAV dynamics are rapid, viral clearance in the early stages of infection, therefore, contributes greatly to limit viral spread. The rapid up-regulation of type I IFN-induced genes is an efficient antiviral barrier that is a crucial first line of host defense (46, 47). These restriction factors intervene in a variety of steps needed in the infection cycle. Surprisingly, we could not detect any signs of priming of the type I IFN host response upon AnxA1 treatment. The lower cytokine titers found in the BAL of AnxA1-treated mice on d 3 upon IAV infection and the concomitant lower expression values of key IFN-induced genes measured in the qPCR also match the lower virus load in the tissues. Interestingly, IFN-induced genes tended toward higher expression in AnxA1-treated animals, which we interpret as a secondary effect resulting from less tissue destruction and, consequently, a higher amount of activated cells. Our findings that AnxA1 did not initially prime or enhance an antiviral response in the target tissue, together with our observation that AnxA1-treated primary lung tissue cells are equally susceptible to PR8 infection compared with control cells, pointed to an immune cell-mediated enhanced viral clearance at early steps in infection, thus limiting viral spread and immune-induced lung injury. A key component of the lung defense against respiratory pathogens is the pool of AMs (33, 34). Remarkably, a higher percentage of these critical and unique macrophages (as identified by the presence of the AM-specific surface marker CD11c and Siglec-F) were already found in the AnxA1-treated mice prior to infection, suggesting that the beneficial effect of administering AnxA1 was due to an expansion of this AM population. Furthermore, these AMs showed enhanced phagocytic activity similar to what has been observed in several studies (48, 49) and thus might eliminate the infectious particles (*i.e.*, act as a sink).

The resident lung phagocytes were long thought to originate from blood monocytes, yet more recent observations strongly point to a self-renewing pool that arises from fetal monocytes shortly after birth (31, 50, 51). In the absence of lung damage, AMs are long-lived and persist for months. Their main function as gatekeepers is seen in removing injured cells and foreign particles without activating an immune response, thus maintaining lung homeostasis and gas exchange (52). Though AMs of noninflamed lungs are in a quiescent state and display only little phagocytic activity sufficient to eliminate the daily amounts of inhaled foreign particles and cellular debris, phagocytosis is up-regulated in the activated AMs (1, 32, 53, 54). The crucial role of AMs in the regulation of IAV infection severity has been established in a number of studies. For instance, the course of IAV infection is much more severe and mortal in mice depleted of AMs, clearly showing the critical role of these immune cells in the protection against IAV-associated pathogenicity (33). Importantly, AMs are significantly depleted during the course of IAV infection, which might contribute to the development of bacterial superinfections, and strategies to counteract AM loss have been discussed as a therapeutic approach in the treatment of respiratory infections (7, 55, 56). However, the mechanisms behind the protective function of AMs in IAV infection are not fully understood and might impact a variety of processes, including AM-mediated tissue repair during respiratory virus infection (9). In addition to clearing necrotic debris, AMs have been described just recently to play a function in reducing the susceptibility of alveolar epithelial cells to IAV infection (33). However, AMs can also be directly infected by IAV (9, 57, 58), although the cycle is abortive and does not sustain the release of progeny. Thus, AMs might act as a sink, thereby limiting IAV spread. Whether all of these protective mechanisms are exerted by AnxA1-expanded macrophages remains to be explored. However, because we found a beneficial effect of the AnxA1 treatment to reduce virus titers at very early stages of the infection, it is highly likely that increased uptake of the incoming viral particles by activated AMs limits the infection.

The lung microenvironment has been shown to continuously supply factors that drive AM maintenance. AM homeostasis and function highly depend on pulmonary GM-CSF expression (59–61). Accordingly, mice deficient in this growth factor (Csf2^−/−^) do not develop AMs (62–64). The much higher severity of IAV infection observed in these mice can be corrected *via* neonatal AM transfer (9), underscoring the vital importance of GM-CSF–dependent AM function in the host defense against IAV (65). Importantly, IAV infection is associated with a prolonged period of GM-CSF deficiency in the lung (66), and lung-specific GM-CSF overexpression 3 d prior to IAV infection is protective and increases recovery of Cd11c^+^ Siglec-F^+^ AMs (67). Indeed, local application of GM-CSF has already been proposed as a therapeutic strategy in pulmonary infections (68, 69). Our observation that AnxA1, *via* FPR2 activation, induces a transient increase in GM-CSF expression associated with a higher number of Siglec-F^+^–positive AMs is well in line with the above data, strongly suggesting that AnxA1 leads to induction of GM-CSF and a concomitant generation of protective AMs (70). This hypothesis is further substantiated by our findings that up-regulation of GM-CSF induced by AnxA1-mediated FPR2 activation is directly associated with AM expansion.

What causes the GM-CSF induction upon AnxA1 treatment? A crucial part of the long-known and well-documented anti-inflammatory effect of AnxA1 and its pharmacophoric N-terminal peptides is based on the functional activation of the FPR subfamily of PRRs (71–73). Though a vast body of evidence confirms that AnxA1 is an endogenous FPR agonist, the specific activation pattern of the individual family members by the full-length protein or peptidomimetics of the N-terminal part is still not completely understood (74). The AnxA1-FPR2 signaling axis is thought to have a predominantly anti-inflammatory role, whereas the FPR1 senses danger signals (including AnxA1 peptides derived from proteolytic cleavage in damaged tissue) and, thus, has a more proinflammatory function (75). Our results, which show that the AnxA1-mediated antiviral protection is conveyed *via* FPR2 but not FPR1 would be consistent with such a possible subfunctionalization of the FPR isoforms, at least in the lung. Interestingly, FPR2 deficiency reduces inflammation in a mouse model of allergic airway disease, associated with less pronounced recruitment of CD11c^+^ dendritic cells (76). Because GM-CSF also stimulates DC activation and proliferation, this is in line with an AnxA1-dependent up-regulation of GM-CSF expression *via* FPR2 activation, such as observed in our study. Contrary to our findings, increased viral replication, associated with more pronounced inflammation and decreased survival, was reported in IAV-infected mice upon FPR2 activation using a synthetic agonist (14, 28, 42, 77). However, the FPR agonist was applied either shortly before (42), simultaneously (28, 42), or after (28) IAV infection in these studies. Therefore, it is unlikely to observe an immunomodulatory effect based on altered differentiation or maturation of the rather slowly proliferating AMs in these experimental setups. The impact of FPR2 activation on IAV infectivity might therefore very much depend on the temporospatial context, with the FPR2 activation prior to infection heightening immune surveillance by expanding the AM pool, whereas FPR2 activity around the time point of infection acts on the epithelium and affects IAV endosomal escape (77).

In summary, we identified a novel role of the AnxA1-FPR2 axis in transiently enhancing the levels of active AMs and showed that AnxA1 treatment prior to IAV infection was beneficial and lung protective. Lung inflammatory injury is exacerbated in AnxA1-deficient mice in several *in vivo* models of lung inflammation (78, 79) and infection (80, 81), suggesting that this proresolving mediator has a general protective role in the lung. However, the molecular mechanisms are still mostly unexplored. Because FPR1-deficient mice still responded to AnxA1, whereas antagonizing FPR2 abolished AnxA1-mediated GM-CSF induction and AM expansion, our data strongly argue for a specific function of the immune-modulatory factor AnxA1 in pulmonary host defense *via* FPR2-dependent signaling.

Infectious diseases are major threats to public health. Both the emergence of new strains and antimicrobial resistance are large and growing problems that require novel treatment strategies. A promising area is host-directed therapies that aim at host factors required for pathogen propagation and host responses to the pathogen rather than directly targeting the pathogen itself (82). Interfering with the host defense system through the use of, for example, endogenous inflammatory mediators is such an emerging novel approach. In this regard, a growing body of convincing studies have identified endogenous specialized proresolving mediators, especially lipid mediators such as lipoxins, resolvins, protectin D1, and maresins, that regulate the termination of inflammation and also function beneficially in infection, pain, and organ protection (83). Of note, proresolving mediators also enhance the host’s ability to clear infections in *in vivo* infection models. This notion was conceptualized by Serhan (83) and confirmed *in vivo* in murine models of bacterial (84) and viral infections (85). Importantly, improved survival and reduced pathology of severe IAV PR8 infection were demonstrated in mice treated with the endogenous lipid mediator protectin D1 before or immediately after virus application (85). Our results show a similar function for the proresolving endogenous protein AnxA1 through its binding to FPR2, which also functions as the receptor for the proresolving lipid lipoxin A4 (86), thus expanding our knowledge on the physiologic function of endogenous proresolving mediators and consolidating FPR2 activation as promising target of a therapy based on this novel concept.

## Abbreviations

AM: alveolar macrophage
AnxA1: annexin A1
BAL: bronchoalveolar lavage
FPR: formyl peptide receptor
GAPDH: glyceraldehyde-3-phosphate dehydrogenase
GM-CSF: granulocyte-M-CSF
IAV: influenza A virus
KO: knockout
LDH: lactate dehydrogenase
MHCCII: major histocompatibility complex class II
p.i.: postinfection
PRR: pattern recognition receptor
qPCR: quantitative PCR

## Supplementary Material

This article includes supplemental data. Please visit *http://www.fasebj.org* to obtain this information.
