# Supplementary material for: The annexin A1/FPR2 signaling axis expands alveolar macrophages, limits viral replication, and attenuates pathogenesis in the murine influenza A virus infection model
Source: FASEB J. 2019 Oct 2;33(11):12188–99. doi: 10.1096/fj.201901265R (PMC6902725; doi:10.1096/fj.201901265R)
Supplement: Supplementary file 1 [file fj.201901265R.sf1.pdf]

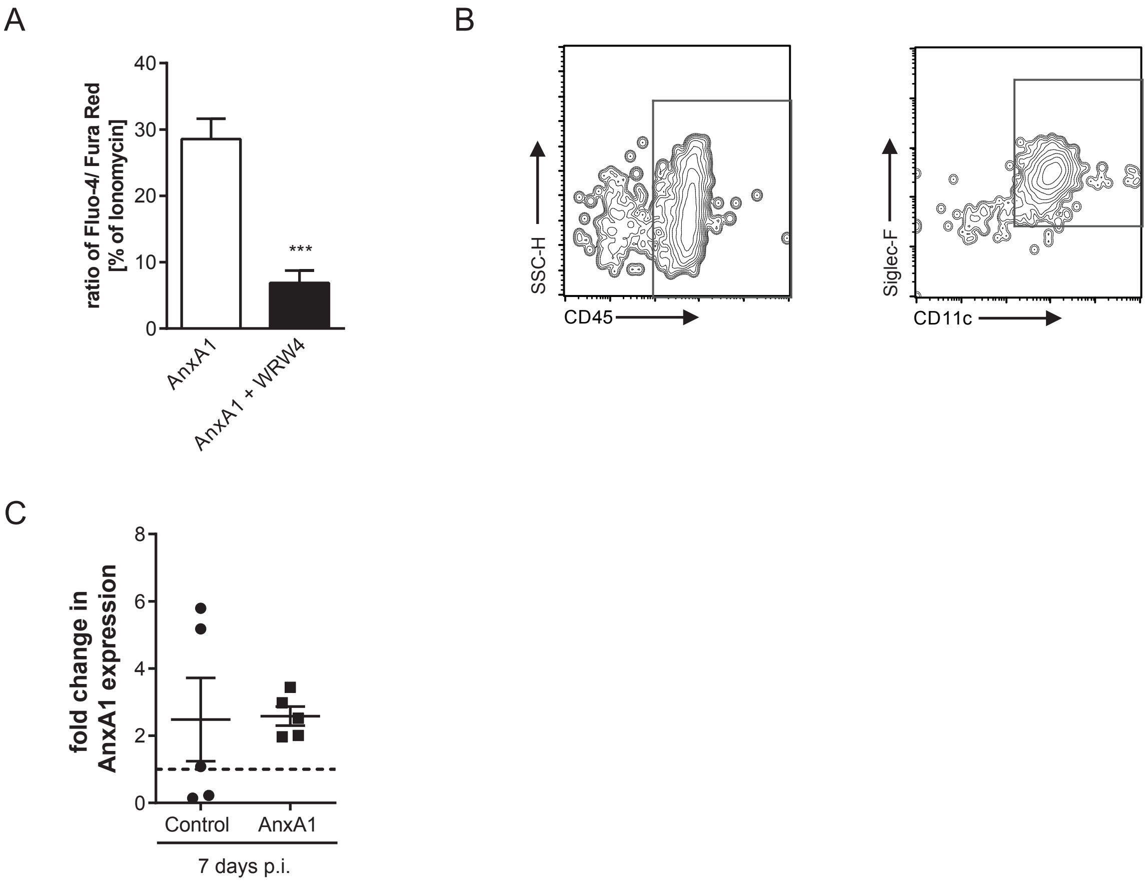

Suppl. Fig. 1. Flow cytometry gating strategy for analysis of BAL immune cell populations. (A) Ratiometric analysis of AnxA1-induced increase in cytosolic Ca^{2+}. (B) BAL cells were gated for size and granularity by forward and side scatter (FSC and SSC), followed by a pre-gating on $CD45^{+}$ events. Cells in BAL fluids of IAV-infected mice obtained at indicated time points of treatment were pre-gated on $CD45^{+}$ events and were analyzed for the levels of AMs ($CD11c^{+}$Siglec-$F^{+}$$MHCII^{+/-}$). (C) qPCR analysis of AnxA1 mRNA levels in lung homogenates of AnxA1 or control-treated mice at day 7 p.i.
